# Reconstructive Surgery in the Elderly: A Case Report on Maintaining the Quality of Life in a Patient with Vulvar Dermatofibrosarcoma Protuberans

**DOI:** 10.3390/diseases12120299

**Published:** 2024-11-22

**Authors:** Marcin Jozwik, Katarzyna Bednarczuk, Zofia Osierda, Maciej Jozwik

**Affiliations:** 1 Department of Gynecology and Obstetrics, Collegium Medicum, University of Warmia and Mazury in Olsztyn, 10-045 Olsztyn, Poland; 2 Scientific Circle of the Department of Gynecology and Obstetrics, Collegium Medicum, University of Warmia and Mazury in Olsztyn, 10-045 Olsztyn, Poland; katarzyna.bednarczuk@student.uwm.edu.pl (K.B.); zofia.osierda@student.uwm.edu.pl (Z.O.); 3 Department of Gynecology and Gynecologic Oncology, Medical University of Białystok, Skłodowskiej Str. 24A, 15-276 Białystok, Poland; maciej.jozwik@umb.edu.pl

**Keywords:** dermatofibrosarcoma protuberans, vulva neoplasm, surgical flap

## Abstract

Background: Dermatofibrosarcoma protuberans (DFSP) is a rare proliferative condition representing skin sarcomas known to locally recur yet very rarely known to metastasize. A typical characteristic is trunk localization, making vulvar occurrences an exception. Complete resection is the primary treatment. Case Report: We present an unusual case of twice-recurrent vulvar DFSP in a 92-year-old woman. Wide local excision with tumor-negative margins (R0 resection) of a grapefruit-sized mass was followed by a rhomboid skin flap transplantation to address a substantial skin deficit at the post excision site. At 14-month follow-up, no signs of recurrence were present, all wounds were healed, and the esthetic results were assessed as highly adequate. Conclusions: This study is an argument for the role of reconstructive surgery in patients of advanced age undergoing oncological procedures; skin transplantations should be considered regardless of the patient’s age. We also provide an updated review on vulvar DFSP.

## 1. Introduction

Dermatofibrosarcoma protuberans (DFSP) is an uncommon skin and/or soft tissue malignancy known to locally recur yet very rarely known to metastasize. Usually, it is a slow-growing, firm, multinodular lesion. When borne in the skin, it usually stems from the dermis and subcutaneous tissue, but not from the epidermis. As a soft tissue tumor, it may involve subcutaneous fat or connective tissue [1,2]. Eisen and Tallini state that DFSP originates in the reticular dermis [3]. Fibroblasts or histiocytes are believed to be the cells of its origin, yet the neoplasm was frequently referred to as a fibrohistiocytic tumor without defining the precise histogenesis [3,4,5,6,7,8,9]. Other likely precursors are perineural cells [10,11]. It was first recognized as a separate entity independently by Sherwell and Taylor in 1890 and later described in much detail by Darier and Ferrand in 1924; nonetheless, the full term ‘dermatofibrosarcoma protuberans’ was proposed by Hoffmann in 1925 [12,13,14,15]. The 2012 guideline by the National Comprehensive Cancer Network states that DFSP is an uncommon, low-to-intermediate-grade sarcoma of fibroblast origin [16].

The incidence of DFSP is low. Yet, even if infrequent, DFSP represents the most common dermal sarcoma (about 1% of all soft tissue sarcomas), more than 1% of all head and neck malignant tumors, and 7% of all head and neck sarcomas [17,18], making the vulvar presentation a rarity with fewer than 100 cases described in the literature to date. Information on incidence patterns of DFSP is still somewhat limited. In a series of 240 patients from the Memorial Sloan-Kettering Cancer Center, New York, NY, USA, there was equal distribution between men and women [19]. Population-based data from the Surveillance, Epidemiology, and End Results (SEER) Program for 1973 to 2002 showed that overall annual incidences of DFSP in the United States were 4.2 per million inhabitants. [20]. Data from other SEER Program registries reviewed for the period from 1992 through 2004 demonstrated the overall DFSP incidence of 4.5 per million inhabitants per year [21]. This relative rarity of DFSP is reflected in the lack of prospective scientific evidence.

We report on a 92-year-old female in good general condition with locally (re-)recurrent DFSP, successfully managed using a well-established approach [22,23]—a wide local excision (WLE) followed by a rhomboid skin flap transplantation. In this case, no postoperative radiation therapy was applied. There were no treatment-related cosmetic or functional issues observed. Our patient is another example of maintaining quality of life and functionality in a patient of a particularly advanced age.

## 2. Case Study

In July 2023, a 92-year-old woman (gravida 2, para 2) presented to the gynecologic oncology outpatient department for consultation regarding a vulvar mass that had reappeared six months earlier and grew quite quickly since then. The patient was normosthenic, not cognitively impaired, and had good control of her long-term arterial hypertension, managed effectively by her family doctor. Her vulvar problem appeared first in 2011 when she underwent a WLE for an 8 × 9 cm mass, and the anatomopathological diagnosis of DFSP was established. She was asymptomatic for another 6 years, and a local de novo growth of 10 × 7 cm in size was observed in 2017. Again, it was managed with complete local resection, and a microscopic diagnosis of DFSP was reconfirmed. During both these procedures, inguinal lymphadenectomies were not performed, as radiologically local lymphatic drainage remained unaffected. Asked about the noticeable sizes of those tumors, the patient explained that they were completely painless and therefore evoked a certain tolerance in her. Her both previous postoperative anatomopathological examinations described a DFSP of typical appearance with low mitotic indices as judged by low Ki67/MIB1 staining in immunohistochemistry.

**Figure 1:**
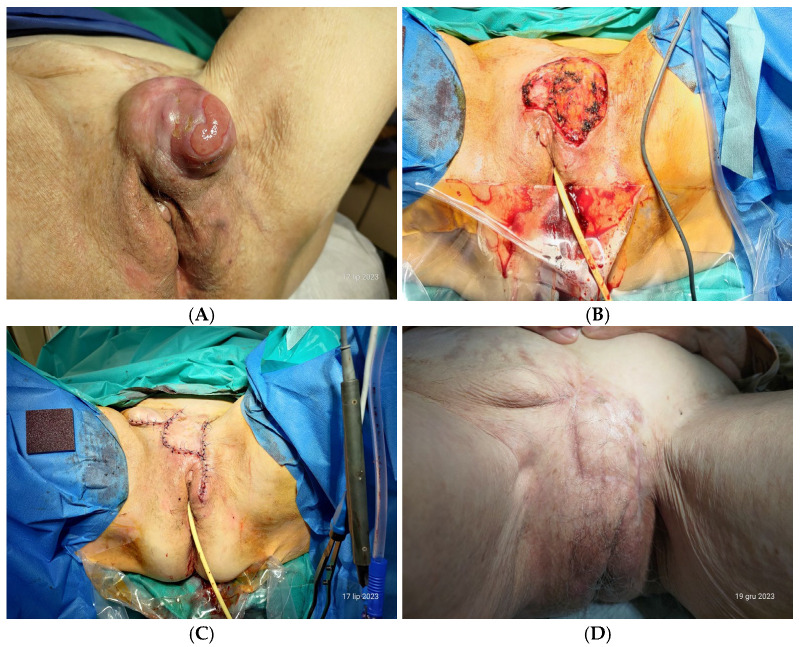
(**A**) Clinical presentation of the second recurrence of DFSP in a 92-year-old woman. (**B**) The intraoperative situation following wide local excision, or WLE. (**C**) The immediate postoperative appearance of skin closures. (**D**) The appearance of the healed hypogastric and vulvar skin at the 5-month follow-up.

A gynecological examination revealed a grapefruit-sized (10 × 9 cm) nodular tumor located between the upper border of her mons pubis, left labium, and left inguinal ligament, with a reduced access to the vaginal introitus (Figure 1A). Extensive skin scarring above the mass was noted. No inguinal lymphadenopathies were detected. The tumor had a somewhat uneven surface but had well-defined borders, was painless at palpation, and presented a small elliptical skin erosion on its left aspect. Yet this erosion did not bleed.

Recurrent disease is surgically more challenging because one often observes fixation of the tumor to deeper structures [24], such as fascia. This is so because previous surgeries usually excised most of the topical subcutaneous tissue. In our patient, the tumor mass was still slightly mobile in reference to the surrounding tissues. Her preoperative A-P chest radiograph showed no suspicious pulmonary opacities, and her abdominal ultrasound was normal. A careful evaluation by palpation of her nodal status, as well as MRI scan, was negative. All in all, the process of medical decisionmaking in her case was based on the following premises: her substantially advanced age; non-fibrosarcomatous DFSP with low mitotic indices in postoperative examinations from her first DFSP surgery and from her first recurrence surgery; actual recurrence not exceeding 10 cm in size (Figure 1A), no signs of local or distant metastatic disease; preserved mobility of the well-circumscribed lesion at palpation; no weight change over the past few months; lack of history of metabolic conditions directly affecting wound healing (such as poorly controlled diabetes mellitus or obesity); and a likely skin deficit following the planned surgery due to previous extensive excisions.

In July 2023, another WLE was performed. A 20-French Foley catheter was placed preoperatively in the urinary bladder. The vulvar skin was incised starting from the mons pubis bilaterally around the lesion. Gross margins of at least 3.0 cm of healthy tissue from the mass were carefully maintained, this based on research that in such instances truly safe, histologically defined margins average 1.6 cm [25]. Moreover, it has been demonstrated that adequate surgical margins as a single modality were associated with 100% local control in all primary DFSPs [26]. Remnants of adipose tissue under the lesion were dissected, and three separate vascular pedicles nourishing the mass were closed and resected with bipolar coagulation (Figure 1B). Intraoperatively, tumor-negative margins (R0 resection) were confirmed. The final specimen was submitted to histopathological examination, and a diagnosis of recurrent DFSP without fibrosarcomatous change was made.

As anticipated, the extent of the wound area after WLE required covering it with a skin graft. To loosen skin tension around the wound, skin mobilization was first performed. The lower abdominal rhomboid skin flap was then raised from the mons pubis skin, preserving the blood supply from abdominal branches of the left superficial epigastric and pudendal arteries. The flap was transposed and secured with resorbable polyglactin No. 1 sutures. The edges of the skin were closed tension-free with non-resorbable polyamide No. 2/0 Blair–Donati stitches for cosmesis. After the procedure, the vaginal introitus regained its symmetry (Figure 1C).

The patient has been followed up for 14 months. At the 5-month checkup, she was well, and the vulvar region and inguinal areas were unsuspected. A transvaginal ultrasound exam showed a small retroverted uterus, a minimal-width endometrium, and atrophied ovaries. No signs of recurrence were noted. The patient expressed her satisfaction with the anatomic results. The esthetic results were assessed as highly adequate (Figure 1D); on the Visual Analog Scale, the patient gave a score of 8 (possible rating: 0–10), and the operating surgeon (first author) gave 7 points.

## 3. Discussion

Our patient is likely to be the oldest case of recurrent vulvar DFSP reported in the literature. Earlier, Soltan described an 83-year-old patient [27], whereas Edelweiss and Malpica reported on a 76-year-old one [1]. However, the dimensions of the presented patient’s tumor, although substantial, were not extreme. The largest lesion (of unknown location) was reported in the Navarrete-Dechent study as having a diameter of 49 cm [28]. In relation to the specific site, the largest so far and, in fact, giant vulvar DFSPs were reported in a Serbian (18 × 10 × 8 cm) and an American (20 × 16 × 13 cm) study [29,30]. As mentioned [19], vulvar location is rare for DFSP (less than 2% of the cases). There have been approximately 60 such women described until 2019 [29]. The largest published series coming from singular institutions were from the M.D. Anderson Cancer Center, Houston, TX, USA, (13 vulvar DFSPs) [1], and from the Mayo Clinic, Rochester, MN, Brigham and Women’s Hospital, Boston, MA, and The Johns Hopkins Hospital, Baltimore, MD combined (11 vulvar DFSPs) [31]. We identified many other, mostly singular, cases of vulvar location reports [27,29,30,31,32,33,34,35,36,37,38,39,40,41,42,43,44,45,46,47,48,49,50,51,52,53], and two reviews [54,55]. The available data from the literature on 43 cases of vulvar DFSP treatment modalities and results have been summarized in Table 1. Consequently, our current count of vulvar DFSPs is 94 reported patients; thus, they are not so exceedingly rare.

The message from the reviews can be summarized as follows. From the Nguyen reappraisal of 54 patients, the tumor histology was uniformly consistent with typical, i.e., low-to-intermediate-grade DFSP, the site of the labia majora was the most commonly affected (53.7%), lesions showed slow to minimal change in size, and no associated lymphadenopathies were reported [54]. In the Mancari report on 68 patients, the indolent nature of the lesion was observed, and the wide use of WLE and partial or total vulvectomy was confirmed [55]. Bertolli et al. applied the complete circumferential peripheral and deep margin assessment, or CCPDMA, technique to remove a vulvar tumor [43]. One modern and attractive alternative to a highly mutilating radical vulvectomy is WLE, which offers the removal of a vulvar tumor with a necessary margin of healthy tissue. In cases of large-sized tumors, however, the excision may leave a significant open wound area. Thus, large postoperative skin defects may result in impaired wound healing, skin tension, and psychological and sexual problems. In particular, the apparent proximity of the anus to the vulvar area carries an increased risk of complications including wound infections [56]. The WLE technique for vulvar DFSP removal provides rather safe, large, negative surgical margins, whereas Mohs micrographic surgery, or MMS, may be considered in exposed areas for its precision and limited cosmetic damage [37,57]. Surgical results may be further improved by postoperative radiotherapy [58,59], which may also be indicated in cases with positive margins or in inoperable patients. Based on recent advancements in cancer genetics, COL1A1-PDGFB gene mutations have been revealed in DFSP. This has resulted in a new approach to DFSP treatment with imatinib methylate, an αPDGFR-targeted therapy for unresectable or metastatic tumors [60].

As with other locations, rare fibrosarcomatous transformation and lung metastases from vulvar DFSPs have been reported [1,45,51]. The patient of Neff et al. had left inguinal lymph node involvement, or local disease spread [30]. Therefore, the evaluation of oncologic risk factors in our patient was mandatory. Advanced age, relatively quick recent growth, and large recurrent tumor size were considered risk factors for potential sarcomatous change. Yet the intraoperative verification of the tumor margins’ negativity (R0 resection) was reassuring. With a history of repeated excision of a large area of hypogastric skin, we focused on the proper covering of the resultant skin defect. Papers by Caretto et al., Lee et al., and Lazarro et al. were all studied, and the rhomboid skin flap was chosen for repair [56,61,62]. Skin flap transplantations in mature patients have been reported to provide good results with no local discomfort with normal activities, including sexual activity, and pleasing esthetic outcome [61,62].

## 4. Conclusions

Vulvar DFSPs are known to locally recur yet very rarely known to metastasize. Usually, a DFSP is a slow-growing, firm, multinodular lesion. Wide radial surgical excision with at least 3 cm margins is the gold standard curative treatment for a localized DFSP but may result in functional impairment or cosmetic disfigurement. We presented a case of twice-recurrent vulvar DFSP in a patient of a highly advanced age, which required the advent of the lower abdominal rhomboid skin flap to cover a skin defect resulting from complete tumor resection. We propose that skin flap transplantations should be considered regardless of the patient’s age.

## Figures and Tables

**Table 1:** Cumulative chronologic data from the literature on vulvar DFSP cases, treatment modalities, and results. NED, no evidence of disease; NIG, no information given; WLE, wide local excision.

Publication	Cases	Patient’s Age (yrs.)	Location	Tumor Size (cm)	Surgical Treatment	Follow-Up (Months)	Recurrence	Outcome
Soltan, (1981) [27]	1	83	left labia minor and major	8.5 × 3.5	excisional biopsy	6	no	NED
Agress et al. (1983) [35]	1	61	upper left labium major and mons pubis	4 × 8	WLE, followed by radical vulvectomy	65	after 5 years and later after 5 months	NED
Bock et al. (1985) [36]	1	52	mons pubis	8 × 8 × 2	WLE	6	no	NED
Barnhill et al. (1988) [37]	1	45	anterior right vulva lateral to the clitoris.	1 × 1	WLE, followed by radical hemivulvectomy with removal of the clitoris.	54	after 3 years	NED
De Chadarévian et al. (1993) [32]	1	1	left labium	8 × 2.5	undefined excision	29	after 12 months	NED
Panidis et al. (1993) [38]	1	30	right labium major	1 × 2	WLE, followed by total vulvectomy	6	after 6 months	NIG
Karlen et al. (1996) [53]	1	36	vulva	5	WLE	27	no	NED
Soergel et al. (1998) [51]	1	47	left labia	3 × 3 × 3	local excision followed by partial radical vulvectomy, followed by WLE	21	3 occurrences of DFSP over approximately a 3-year period, later after 16 months, and after the next 5 months	metastatis
Ghorbani et al. (1999) [39]	4	36–69 (mean 54)	left paraclitoral area (1 case), right labium major (1 case), left labium major (1 case), and mons pubis (1 case)	1.2–5.0	WLE (3), refused (1)	240	3	NED (2 cases), alive with disease (1 case), dead of other causes (1 case)
Moodley & Moodley (2000) [50]	1	39	left labium major	8 × 12	reexcision with 3 cm lateral margin and depth down to the pubic periosteum and fascia of the urogenital diaphragm	3	no	NED
Vanni et al. (2000) [33]	1	39	centered in the vulva, extending to the perineal raphe	6 × 6	WLE	29	no	NED
Ohlinger et al. (2004) [40]	1	36	left side of the vulva	2.3 × 2.8	excisional biopsy	108	5 recurrences	complete tumor clearance was achieved only after 5 additional surgeries.
Olejek et al. (2008) [41]	1	49	vulva	n/a	n/a	n/a	n/a	NIG
Oge et al. (2009) [42]	1	56	vulva	3 × 3	wide and deep local excision	15	recurrence	NED
Edelweiss & Malpica (2010) [1]	13	23–76 (mean 46)	right labium major (6), right labium major/crural fold (1), right labium major/mons pubis (1), left labium major (2), paraclitoral (2), and mons pubis (1)	1.2 to 15 (median, 4)	excisional biopsy (3), excisional biopsies followed by WLE (7), WLE (2), excisional biopsy followed by radical vulvectomy (1)	2 to 444	7	alive with disease (2), dead of other causes (1), dead of disease (1), NED (9)
Zizi-Sermpetzoglou et al. (2012) [48]	1	66	mons pubis	8 × 5	WLE	n/a	n/a	NED
Bertolli et al. (2014) [43]	2	28, 57	mons pubis (1), right labium major (1)	5 × 5, 2 × 2	partial vulvectomy with clitoris preservation (1), complete circumferential and peripheral deep margin assessment and WLE (1)	40 and 10	no (2)	NED (2)
Gilani & Al-Khafaji, (2014) [44]	1	61	left mons pubis	N/A	excisional biopsy and 2 subsequent resections	12	no	NED
Wiszniewska et al. (2016) [49]	1	44	right labia	5.0 × 3.5 × 3.0	wide excision with positive margins and subsequent re-excision with negative margins	18	no	NED
Vathiotis et al. (2018) [45]	1	72	right labium major	N/A	WLE	48	yes	lung metastasis
Jeremic et al. (2019) [29]	1	55	mons pubis, clitoris and upper half of labia majora	18 × 10 × 8	radical tumor excision followed by soft tissue defect reconstruction	18	no	dead of other causes
Neff et al. (2019) [30]	1	57	mons pubis and left labium major	20 × 15	radical vulvectomy with vertical rectus abdominis myocutaneous flap	12	no	NED
Zemni et al. (2019) [46]	1	47	left labium major	5 × 4 × 3.5	WLE	1	no	NIG
Goyal et al. (2021) [47]	1	35	upper part of right labium major	5 × 6	WLE	24	2 recurrences	NED
Algarra-Sahuquillo et al. (2024) [34]	3	44, 49, 13	right labium major (1 case), left labia major and minor, clitoris (1 case), and pubis (1 case)	2 × 1; 6 × 4.5; 6 × 5	Slow Mohs Micrographic Surgery	171; 67; 67	no	NED

## Data Availability

All research data are available upon request from the corresponding author.
